# Racial, Ethnic, and Gender Representation in Leadership Positions at National Cancer Institute–Designated Cancer Centers

**DOI:** 10.1001/jamanetworkopen.2021.12807

**Published:** 2021-06-07

**Authors:** Austin Morgan, Kanan Shah, Kevin Tran, Fumiko Chino

**Affiliations:** 1 University of Arkansas for Medical Sciences, Little Rock; 2 New York University Grossman School of Medicine, New York; 3 University of Arkansas, Rogers; 4 Department of Radiation Oncology, Memorial Sloan Kettering Cancer Center, New York, New York

## Abstract

**Importance:**

Increasing diversity is beneficial for the health care system and patient outcomes; however, the current leadership gap in oncology remains largely unquantified.

**Objective:**

To evaluate the gender, racial, and ethnic makeup of the leadership teams of National Cancer Institute (NCI)-designated cancer centers and compare with the city populations served by each center.

**Design, Setting, and Participants:**

This retrospective cross-sectional study examined gender, race, and ethnicity of leadership teams via publicly available information for NCI-designated cancer centers and compared results with national and city US census population characteristics, as well as active physician data. Data were analyzed in August 2020.

**Main Outcomes and Measures:**

Racial, ethnic, and gender diversity (identified via facial recognition software and manual review) of leadership teams compared with institution rank, location, team member degree(s), and h-index.

**Results:**

All 63 NCI cancer centers were included in analysis, and all had identifiable leadership teams, with a total of 856 members. Photographs were not identified for 12 leaders (1.4%); of the remaining 844 leaders, race/ethnicity could not be identified for 7 (0.8%). Women make up 50.8% of the US population and 35.9% of active physicians; in NCI cancer centers, 36.3% (306 women) of cancer center leaders were women. Non-Hispanic White individuals comprise 60.6% of the US population and 56.2% of active physicians, but 82.2% of cancer center leaders (688 individuals) were non-Hispanic White. Both Black and Hispanic physicians were underrepresented when compared with their census populations (Black: 12.7% of US population, 5.0% of active physicians; Hispanic: 18.1% of US population, 5.8% of active physicians); however, Black and Hispanic individuals were even less represented in cancer center leadership positions (29 Black leaders [3.5%]; 32 Hispanic leaders [3.8%]). Asian physicians were overrepresented compared with their census population (5.6% of US population, 17.1% of active physicians); however, Asian individuals were underrepresented in leadership positions (92 Asian individuals [11.0%]). A total of 23 NCI cancer centers (36.5%) did not have a single Black or Hispanic member of their leadership team; 8 cancer centers (12.7%) had an all non-Hispanic White leadership team. A multivariate model found that leadership teams with more women (adjusted odds ratio, 1.73 [95% CI, 1.02-2.93]; *P* = .04) and institutions in the South (adjusted odds ratio, 2.31 [95% CI, 1.15 to 4.77]; *P* = .02) were more likely to have at least 1 Black or Hispanic leader. Pearson correlation analysis showed weak to moderate correlation between city Hispanic population and Hispanic representation on leadership teams (*R* = 0.5; *P* < .001), but no significant association between Black population and Black leadership was found.

**Conclusions and Relevance:**

This cross-sectional study found that significant racial and ethnic disparities were present in cancer center leadership positions. Establishing policy, as well as pipeline programs, to address these disparities is essential for change.

## Key Points

**Question:**

What is the diversity of the leadership teams of National Cancer Institute–designated cancer centers, and how does this compare with the populations served by each center?

**Findings:**

In this retrospective cross-sectional study including 63 cancer centers with 856 leadership team members, non-Hispanic White men were disproportionately represented in leadership while Black, Hispanic, and Asian leaders were underrepresented. Centers with more women leaders were more likely to have at least 1 Black or Hispanic leader; however, diverse cities were not necessarily more likely to have representatively diverse leaders.

**Meaning:**

These findings suggest that establishing policy and pipeline programs to address significant racial and ethnic disparities in cancer care leadership positions is crucial for change.

## Teaser

This cross-sectional study examines racial, ethnic, and gender representation in leadership positions at National Cancer Institute (NCI)–designated cancer centers.

## Introduction

Racial disparities in cancer care access, delivery, and outcomes are well documented.^1^ They are, in part, due to lack of access to high-quality, culturally competent care.^2^ An increasing body of research indicates that increasing diversity among health care leaders and physicians is beneficial for the health care system and patient outcomes.^3,4^ One 2017 study^5^ found that older patients treated by women internists had lower mortality and readmissions compared with those treated by men internists, and a 2020 study^6^ found that newborn mortality in Black infants was halved when they were cared for by a Black physician. When patients are treated by racially and ethnically concordant physicians, they are more likely to receive necessary medical care, including preventative health care.^7^

Specifically within cancer care, physician diversity is essential in the provision of high quality cancer treatment to increasing racial/ethnic minority communities, such as Hispanic communities, who have made up more than half of the population growth in the US.^8^ Implicit bias has known negative outcomes within oncology,^9^ and improving diversity can lead to increased intercultural responsiveness and foster trust and comfort for patients.^10^ Developing an oncology workforce that reflects the patients whom it serves has been a priority for both American Society of Clinical Oncology^11^ and the National Cancer Institute (NCI),^12^ as there are known gender and racial/ethnic gaps within the physician pipeline and workforce.^13,14^ However, the leadership gap in cancer care remains largely unquantified and is a key component to understanding how institutions may prioritize equity, diversify hiring, and promote systemwide change to improve cancer disparities. This cross-sectional study was designed to evaluate the gender, racial, and ethnic makeup for the full leadership team of NCI-designated cancer centers and to compare this with the diversity of actively practicing physicians and with the city populations served by each center.

## Methods

This cross-sectional study was reviewed by the institutional review board of Memorial Sloan Kettering and was found to not meet the definition of human participants research; therefore, this study did not require oversight or informed consent. This study followed the Strengthening the Reporting of Observational Studies in Epidemiology (STROBE) reporting guideline for reporting observational cross-sectional studies.

The names, photographs, degrees, academic titles, and h-index scores of leadership team members were obtained via publicly available information for each of the 63 NCI-designed cancer treatment centers. Leadership team members are defined as individuals who were identified on each cancer center’s respective website as “leaders,” part of the “leadership team,” or listed under the center “leadership” page. The h-index is a measure of a scholar’s productivity and publication impact; it is calculated as the highest number of highest cited papers. It was captured as a measure of leadership academic productivity.

Gender, race, and ethnicity were determined by facial recognition software (Kairos) with secondary manual review and additional classification from first and last name and biography for ambiguous identities. Manual review was performed by A.M. and F.C. Both authors individually reviewed each photograph and name and compared that to the facial recognition algorithm output. Any disputes or ambiguous photographs required further inspection of the individual’s affiliations; unresolved disputes were marked as unknown race or ethnicity. The facial recognition algorithm used was developed from a large diverse database; the algorithm was specifically designed to reduce known bias apparent within other software.^15^

City population demographic information was collected from the US Census,^16^ and centers were grouped geographically (Northeast, South, West, Midwest). Cancer center rank was identified from US News and World Report 2020 Rankings.^17^ Information on actively practicing physicians in the United States was gathered from the 2019 Association of American Medical Colleges workforce data.^18^

### Statistical Analysis

Pearson correlation and multivariate analysis were used to assess the association between racial/ethnic representation on leadership teams and location of institution, institution ranking, median h-index of researchers on leadership team, and composition of leadership team with regards to sex and degrees earned using RStudio statistical software version 1.2.5033 (R Project for Statistical Computing). Data were analyzed in August 2020. All *P* values were from 2-sided tests, and the results were deemed statistically significant at *P* < .05.

## Results

All 63 NCI cancer centers had identifiable leadership teams, with a total of 856 leadership members. Leadership teams ranged from 1 to 42 members. We were unable to obtain photographs for 12 leaders (1.4%); of 844 remaining leaders, facial recognition software and manual review were unable to identify race/ethnicity of 7 leaders (0.8%). Of 844 leaders with photographs, we were able to identify gender for all of them, and 306 (36.3%) were women. Of 837 leaders with photographs who could be assigned race/ethnicity, 688 (82.2%) were non-Hispanic White individuals, 29 (3.5%) were Black individuals, 92 (11.0%) were Asian individuals, and 32 (3.8%) were Hispanic individuals.

**Figure 1.**
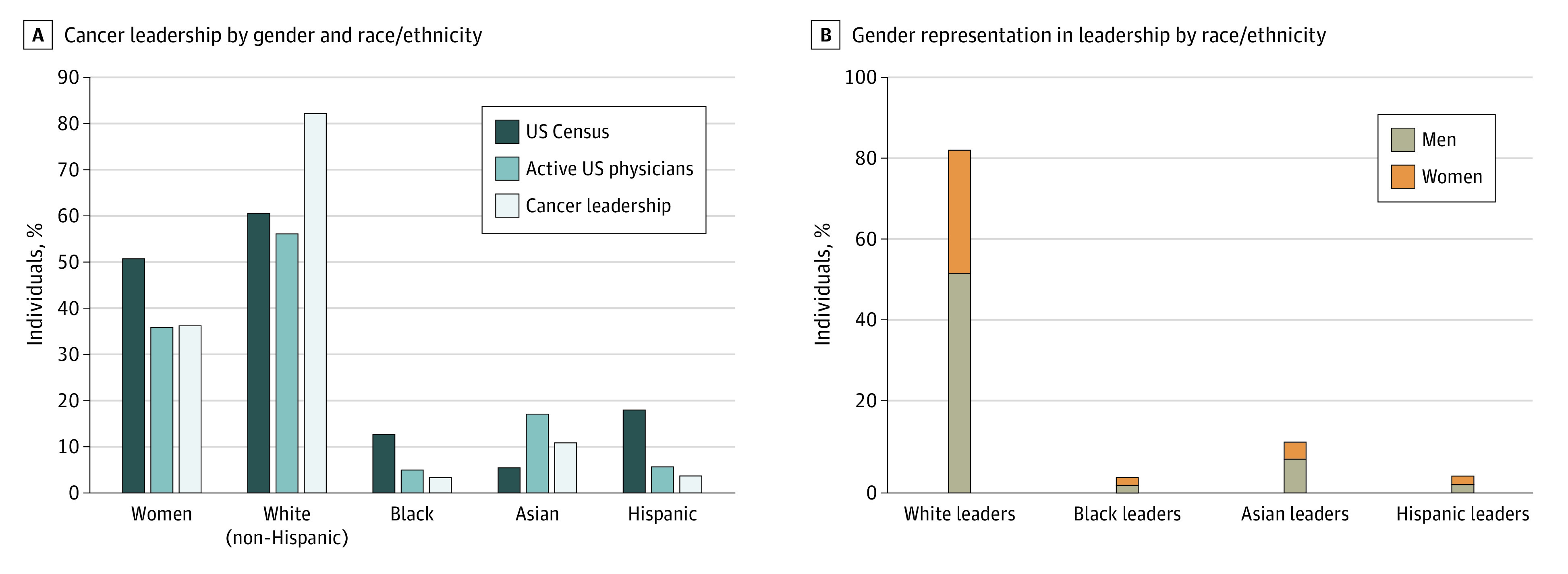
Gender, Race, and Ethnicity in Cancer Leadership Population data from the US Census, and active physician data are from the Association of American Medical Colleges.

Comparisons between the gender, racial, and ethnic make-up of the entire US population, active physicians, and physicians in cancer center leadership roles are shown in Figure 1. Women are approximately half of the US population (50.8%), but only about one-third of active physicians (35.9%) and cancer center leadership members (306 women [36.3%]). Non-Hispanic White people represent less than two-thirds of the US population (60.6%) and slightly more than half of the active physicians (56.2%), but non-Hispanic White leaders make up 82.2% of cancer center leadership (Figure 1). Compared with their census populations, both Black and Hispanic physicians were underrepresented (Black: 12.7% of US population, 5.0% of active physicians; Hispanic: 18.1% of US population, 5.8% of active physicians); however, they were even more scarce in leadership positions (Black leaders, 3.5%; Hispanic leaders, 3.8%) (Figure 1). Asian physicians were overrepresented compared with their census population (17.1% of active physicians, 5.6% of US population); however, Asian individuals were underrepresented in leadership positions (11.0%) when compared with their percentage of active physicians (Figure 1).

Of 63 cancer centers assessed, 23 centers (36.5%) did not have a single Black or Hispanic member of their leadership team; 41 centers (65.1%) did not have a Black leader, and 36 centers (57.1%) did not have a Hispanic leader. Furthermore, 8 centers (12.7%) had an all non-Hispanic White leadership team and 16 centers (25.4%) had 90% or more non-Hispanic White leadership. Of 33 cancer centers in cities with at least 20% Black population, 21 centers (63.6%) did not have a Black leadership team member. Of 26 cancer centers in cities with at least 20% Hispanic population, 8 centers (30.8%) did not have a Hispanic leadership team member.

**Figure 2.**
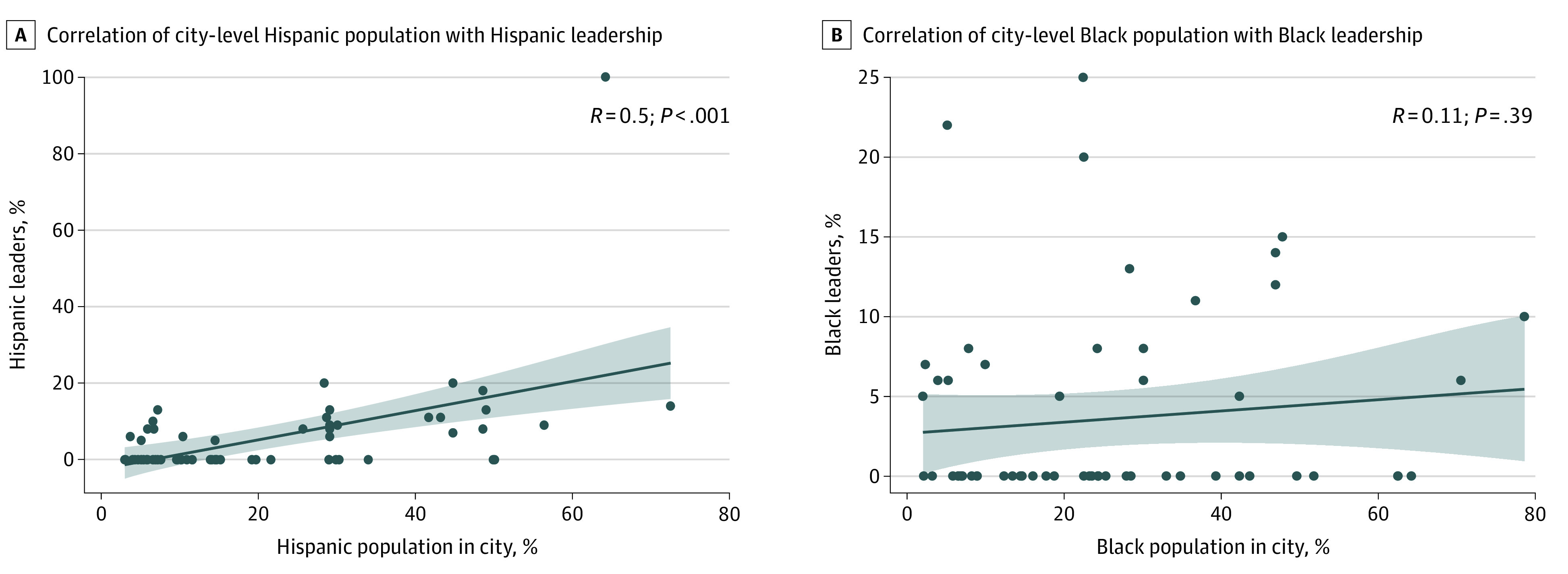
Pearson Correlation Between Diversity of City and Diversity of Leadership Team Line indicates correlation; shaded area, 95% CI; and dots, individual data points.

A multivariable linear regression model assessing the association between percentage of Black or Hispanic leadership team members and team characteristics found a positive correlation with increasing number of MDs (compared with MD/PhDs) on the team (β = 0.23 [95% CI, 0.04 to 0.41]; *P* = .02) and for cancer centers located in the South (β = 10.54 [95% CI, 1.75 to 19.33], *P* = .02). Cancer centers ranked outside of the top 25 were less likely to have a diverse leadership team (β = −8.75 [95% CI, –16.20 to –1.28]; *P* = .02) (Table 1). A multivariate model found that teams with more women (adjusted odds ratio, 1.73 [95% CI, 1.02 to 2.93]; *P* = .04) and institutions in the South (adjusted odds ratio, 2.31 [95% CI, 1.15 to 4.77]; *P* = .02) were more likely to have at least 1 Black or Hispanic leadership member (Table 2). Pearson correlation analysis showed weak to moderate correlation between city Hispanic population and representation on leadership teams (*R* = 0.5, *P* < .001) but no association between Black population and leadership (Figure 2).

**Table 1.** Regression Models for Diversity of Leadership Team

Characteristic	% of leadership team who are Black or Hispanic leaders	
	β	*P* value
Initial regression model		
% of team who are men	0.7 (–2.14 to 3.06)	.61
% of team with MD	0.22 (–0.02 to 0.45)	.08
% of team with MD/PhD	–0.08 (–0.46 to 0.30)	.67
Institution ranking		
Top 25	1 [Reference]	NA
26-50	–7.22 (–15.12 to 1.00)	.07
>50	–8.55 (–18.39 to 1.83)	.09
Institution region		
Northeast	1 [Reference]	NA
Midwest	2.37 (–7.29 to 12.46)	.63
South	10.46 (1.48 to 20.02)	.02
West	7.16 (–4.58 to 19.78)	.23
Median h-index	0.12 (–0.09 to 0.34)	.25
Final regression model (selection via backward elimination)		
% of team with MD	0.23 (0.04 to 0.41)	.02
Institution ranking		
Top 25	1 [Reference]	NA
26-50	–8.75 (–16.20 to –1.28)	.02
>50	–9.22 (–18.91 to 0.48)	.06
Institution region		
Northeast	1 [Reference]	NA
Midwest	1.99 (7.52 to 11.51)	.68
South	10.54 (1.75 to 19.33)	.02
West	2.90 (–6.54 to 12.33)	.54

Abbreviation: NA, not applicable.

**Table 2.** Regression Models for Diversity of Leadership Team

Characteristic	Any Black or Hispanic leadership team member	
	Adjusted odds ratio (95% CI)	*P* value
Initial regression model		
Gender		
Men	1 [Reference]	NA
Women	1.77 (1.01-3.13)	.04
Region of home institution		
Northeast	1 [Reference]	NA
Midwest	1.30 (0.52-3.15)	.56
South	2.79 (1.29-6.16)	.01
West	1.61 (0.74-3.55)	.22
Degree		
MD	1 [Reference]	NA
MD/PhD	0.58 (0.13-1.79)	.40
PhD	1.08 (0.56-2.11)	.81
RN	0.56 (0.03-3.16)	.59
Other	0.92 (0.42-1.95)	.83
Rank of institution		
Top 25	1 [Reference]	NA
26-50	0.46 (0.21-0.98)	.04
>50	1.09 (0.56-2.11)	.80
Median h-index	0.99 (0.99-1.00)	.04
Final regression model (selection via backward elimination)		
Gender		
Men	1 [Reference]	NA
Women	1.73 (1.02-2.93)	.04
Region of home institution		
Northeast	1 [Reference]	NA
Midwest	0.95 (0.40-2.17)	.90
South	2.31 (1.15-4.77)	.02
West	1.95 (0.92-4.16)	.08

Abbreviation: NA, not applicable.

## Discussion

This cross-sectional study found that White men were disproportionately represented in the highest levels of cancer center leadership while Black, Hispanic, and Asian leaders did not have proportionate space at the decision-making table. Centers with more women leaders were more likely to have diverse leadership teams; however diverse cities were not necessarily more likely to have representatively diverse leaders, with Black leaders rare even in cities with large Black populations. These findings suggest that marginalized racial/ethnic groups, such as Black, Hispanic, and Asian individuals, may face stark challenges limiting their advancement into senior leadership positions.

Our findings are similar to a recent director survey from the Association of American Cancer Institute and *The Cancer Letter*,^19^ which found a high percentage of White men in director roles. NCI-designated cancer centers are the anchors of the nation’s cancer research effort, and center leaders are actively involved in setting the standards of care and the future direction of cancer treatment in the US, including cancer clinical trials. Thus, lack of diversity within these leadership teams is particularly concerning, as they reflect the immediate pipeline of directors, and that indicates that not enough individuals of marginalized racial or ethnic identities are making it into the pipeline.

Improving the diversity of leadership teams is in an important component of improving health equity, as diverse leaders can help reduce the cultural gap. Different backgrounds benefit patients and improve recruitment of future physicians underrepresented in medicine.^20,21^ There are immediate strategies that can be used to improve diversity, including acknowledging that systemic bias is prevalent and investigating the hiring process to see where it lies. Widely promoting open positions and implementing blinded screening policies for new hires can help diversify the hiring pool and avoid the pitfalls of implicit bias during the hiring process. Implicit bias training, diversifying search committees, and implementation of holistic review in the hiring process are also all positive steps to diversify leadership.^20,22^ Additionally, making diversity and health equity a stated goal of the entire health system can focus priorities across the campus on making clear and concrete steps toward improvement. This requires structural change to eliminate barriers toward advancement that many women and individuals from racial and ethnic minority groups have faced, including inadequacy of mentoring, lack of protected time for academic scholarship, and bias in promotion to midlevel leadership positions (eg, department head, chair, or vice chair). The baseline information provided by this study could serve as a comparison for the future to measure how successful diversity initiatives have been.

### Limitations

This study has some limitations, including the categorization of leadership per the US Census, which includes those with Middle Eastern and North African ethnicities as White race. This places some leaders who may have dealt with significant racial/ethnic or cultural bias in a category that may not reflect their contributions to diversity. Additionally, binary male/female gender does not appropriately categorize leaders who may identify as nonbinary or gender nonconforming. Another limitation lies with accurately identifying race and ethnicity from a photograph, name, and biography, as self-reported race/ethnicity continues to be the criterion standard for this information. Facial recognition software has known racial bias and can be ethically dubious in use.^23^ We specifically sought out a software that was created to combat known bias concerns and was developed from a large, international database.^15^ Also the system of facial recognition combined with manual review had a 96.2% concordance to self-report based on prior work by the study team.^24^ Population demographic information was limited to the cities where each cancer center was located; this may not reflect the true demographic characteristics of patients served over the center’s entire catchment area. Additionally, leadership teams found via publicly available information may not be up to date and thus may not reflect their current state of diversity.

## Conclusions

This cross-sectional study found significant racial and ethnic disparities in cancer care leadership positions. Racial/ethnic identities that are underrepresented in medicine were even more underrepresented in leadership; establishing policy and pipeline programs to address these disparities is essential for change. Workforce diversity and inclusion are critical for improving health care equity; hiring and retaining leaders of diverse backgrounds should be a top priority for the future.
